# New combination chemotherapy of cisplatin with an electron-donating compound for treatment of multiple cancers

**DOI:** 10.1038/s41598-020-80876-z

**Published:** 2021-01-12

**Authors:** Qinrong Zhang, Qing-Bin Lu

**Affiliations:** 1 Department of Physics and Astronomy, Faculty of Science, University of Waterloo, 200 University Avenue West, Waterloo, ON N2L 3G1 Canada; 2 Department of Biology and Chemistry, Faculty of Science, University of Waterloo, 200 University Avenue West, Waterloo, ON N2L 3G1 Canada

**Keywords:** Biochemistry, Biophysics, Cancer, Cell biology, Chemical biology, Drug discovery, Molecular biology

## Abstract

Cisplatin is the first and most widely used platinum-based chemotherapy drug and is the cornerstone agent in treating a broad spectrum of cancers. However, its clinical application is often limited by severe toxic side effects and drug resistance. Based on the discovered dissociative electron transfer mechanism of cisplatin, a novel combination of cisplatin with [9-(2-carboxyphenyl)-6-diethylamino-3-xanthenylidene]-diethylammonium chloride (basic violet 10, BV10) is proposed to potentiate the chemotherapeutic effect of cisplatin. Here, we show that this combination enhances the anti-cancer effect of cisplatin in both in vitro cell lines and in vivo xenograft mouse models of cisplatin-sensitive and -resistant lung, ovarian and cervical cancers while introducing minimal additional toxic side effects. Furthermore, femtosecond time-resolved laser spectroscopic measurements demonstrate that cisplatin reacts with BV10 via an electron transfer mechanism. These results indicate that the combination of cisplatin with BV10 is promising for improving the chemotherapy of cancers with various extents of cisplatin resistance.

## Introduction

The discovery of the anti-cancer activity of cisplatin (CDDP) is a milestone in the chemotherapy of cancer^1^. Chemotherapies with platinum-based anticancer drugs are now standard treatments for a broad spectrum of cancers, including ovarian cancer, cervical cancer, lung cancer, head and neck cancer, bladder cancer, and lymphoma^2–4^. Despite its great success as an anti-cancer drug, CDDP has severe toxic side effects and often develops drug resistance with time, which limit its clinical use^5–10^. Its dose-dependent toxicities include nephrotoxicity and neurotoxicity^6,7^, mainly due to the binding of the heavy-metal platinum (Pt) to proteins in kidneys^8,9^. These problems are so severe that they even prompted the call to discontinue the clinical use of Pt-based anticancer drugs^10^.

Combination therapy is one of the efforts to overcome the above problems. By combining multiple drugs, one expects to reduce individual toxicity from each drug; at the same time, cancer cells are less likely to be resistant to multiple drugs simultaneously^11^. Also, by combining multiple drugs targeting different parts of a cancer cell and/or different cell cycles, one also expects to enhance the anti-cancer efficacy. CDDP-based combination chemotherapy has achieved some success in the clinic^2,3,11–16^. In most cases, however, they exhibit additive*,* rather than synergistic, therapeutic effects. Moreover, the use of multiple cytotoxic drugs sometimes induces additive toxicity and even deaths^7,11,16^.

DNA is the major target of CDDP. The anti-cancer activity of CDDP arises from the induced distortion in the structure of DNA, which triggers cell death^17^. CDDP mainly forms intrastrand 1,2-GG adducts (60–65%) and intrastrand 1,2-AG adducts (20–25%)^18,19^. It was long believed that a hydrolysis process of CDDP must occur before it can modify DNA. Based on the hydrolysis mechanism, many studies have aimed to circumvent the drawbacks of CDDP over the past 50 years; over 3000 CDDP analogues have been designed, synthesized and tested, but finally only two have been approved by the FDA to treat certain types of cancer: oxaliplatin and carboplatin. This fact implies that a precise understanding of the molecular mechanism of the cytotoxicity of CDDP was not revealed until the direct observation of the initial chloride-bond breaks in CDDP was made by femtosecond time-resolved laser spectroscopy (fs-TRLS). Using fs-TRLS, Lu et al.^20^ have unraveled the molecular mechanism for enhancing the therapeutic efficacy of radiotherapy by low-dose CDDP, and found that it is due to the extremely effective dissociative electron transfer (DET) reaction of CDDP with the weakly-bound prehydrated electron (${e}_{pre}^{-}$) generated by radiolysis of water:

1a $${\mathrm{e}}_{\mathrm{pre}}^{-}+\mathrm{Pt}{({\mathrm{NH}}_{3})}_{2}{\mathrm{Cl}}_{2}\stackrel{ }{\to }{[\mathrm{Pt}{({\mathrm{NH}}_{3})}_{2}{\mathrm{Cl}}_{2}]}^{*-}\stackrel{ }{\to }\mathrm{Pt}{({\mathrm{NH}}_{3})}_{2}{\mathrm{Cl}}^{\bullet}+{\mathrm{Cl}}^{-}$$

1b $${\mathrm{e}}_{\mathrm{pre}}^{-}+\mathrm{Pt}{({\mathrm{NH}}_{3})}_{2}\mathrm{Cl}\stackrel{ }{\to }{[\mathrm{Pt}{({\mathrm{NH}}_{3})}_{2}\mathrm{Cl}]}^{*-}\stackrel{ }{\to }{\mathrm{Pt}{({\mathrm{NH}}_{3})}_{2}}^{\bullet}+{\mathrm{Cl}}^{-}$$

The resultant cis-Pt(NH_3_)_2_ radical highly effectively leads to DNA strand breaks^20^. Furthermore, it was also shown that for chemotherapy, CDDP indeed preferentially attracts two electrons from two neighboring guanine bases in DNA, since guanine is the most favored electron donor in DNA^21^. This DET mechanism has uncovered the long-existing mystery of why CDDP results in a preferential binding to two neighboring G bases in DNA^21^. Subsequently, this DET mechanism of CDDP has been confirmed both experimentally and theoretically by other researchers^22,23^.

This new mechanistic insight, obtained by the novel femtomedicine approach^24^, has potential to improve existing therapies using CDDP, discover new anti-cancer agents, and enable novel combinations of CDDP for treating challenging cancers. Indeed, the studies in femtomedicine have led to the discoveries of not only the molecular mechanisms of CDDP^20,21^ and reductive DNA damage^25,26^ but the combination therapy of CDDP^27^ and novel anti-cancer agents for targeted chemotherapy^28^ and radiotherapy^29^. This innovative approach may offer an effective and economic strategy to develop novel cancer therapies.

Based on the DET mechanism of CDDP, one of our efforts was to identify effective molecular promoters (PMs) to enhance the cytotoxicity of CDDP and to overcome the drug resistance, thus widening the clinical application of CDDP. It is expected that the DET reaction of a PM molecule as an electron-donating agent with CDDP, preferentially occurring inside the cancer cell, will generate the reactive radical and thus enhance the cytotoxicity of CDDP:

2 $$\mathrm{PMx}+\mathrm{Pt}{({\mathrm{NH}}_{3})}_{2}{\mathrm{Cl}}_{2}\stackrel{ }{\to }{{\mathrm{PMx}}^{+}+[\mathrm{Pt}{({\mathrm{NH}}_{3})}_{2}{\mathrm{Cl}}_{2}]}^{*-}\stackrel{ }{\to }{\mathrm{PMx}}^{+}:{\mathrm{Cl}}^{-}+\mathrm{Pt}{({\mathrm{NH}}_{3})}_{2}{\mathrm{Cl}}^{\bullet}$$

The resultant Pt(NH_3_)_2_Cl^•^ or Pt(NH_3_)_2_^•^ (by further reaction with another PM molecule) radical can lead to DNA strand breaks^20^, adding to the intrastrand cross-links caused by CDDP alone.

We previously studied the combination of CDDP and a well-known biochemical electron donor tetramethyl-*p*-phenylenediamine (TMPD) and have obtained encouraging in vitro results of improving the therapeutic efficacy of CDDP^27^. However, TMPD itself not only shows some toxicity but is extremely easy to auto-ionize/oxidize in water and thus to lose its electron-donating capability, leading to its limited in vivo efficacy in enhancing the cancer therapy with CDDP. Here, we report a novel combination therapy of cisplatin (CDDP) with a new PM, Rhodamine-B (RDM-B or BV10), to overcome this barrier for potential clinical use. We present both effective in vitro and in vivo results of this combination in treating lung, ovarian and cervical cancers with various extents of CDDP resistance. Moreover, our fs-TRLS measurements provide spectroscopic evidence of the DET reaction mechanism of this novel combination.

## Results

### DNA double-strand break (DSB) measurements by agarose gel electrophoresis and γH2AX labeling

The enhancement factor in CDDP-induced DNA DSBs by a PM is an important indicator in evaluating the efficacy of the PM-CDDP combination. The DET-based combination is expected to enhance anti-cancer effect of CDDP by generating more DNA-damaging radicals ($\mathrm{Pt}{({\mathrm{NH}}_{3})}_{2}{\mathrm{Cl}}^{\bullet}$ and/or $\mathrm{Pt}{{({\mathrm{NH}}_{3})}_{2}}^{\bullet}$) with the combination of BV10, as expressed in reaction (2). Thus, we measured the DSB yields of both plasmid DNA and cellular DNA induced by CDDP only and its combination with RDM-B (BV10), by agarose gel electrophoresis and γH2AX labeling, respectively.

**Figure 1:**
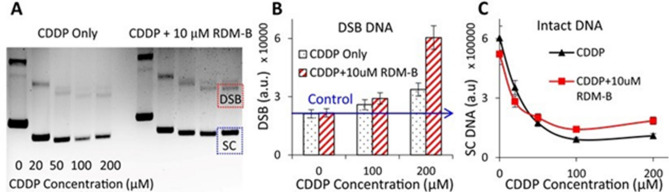
DNA damage measurements using agarose gel electrophoresis. (**A**) Gel images of plasmid DNA treated with various concentrations of CDDP with/without the presence of 10 μM RDM-B (BV10), where the three separate bands from top to bottom represent DNA with single-strand break (SSB), double-strand break (DSB, red dashed square), and intact supercoiled DNA (SC, blue dashed square). The yields of DSB DNA and the amount of SC DNA are shown in (**B**) and (**C**), respectively.

The agarose gel images and the DSB yields of plasmid DNA treated with various concentrations of CDDP and its combination with 10 μM BV10 for 150 min at 37 °C are shown in Fig. 1A. As seen from Fig. 1B, the combination of CDDP and BV10 increased the yield of DNA DSBs by a factor of approximately 3 in comparison with the treatment of CDDP only. Moreover, it is notable that the amount of ‘intact’ plasmid DNA, i.e., the intensity of the band corresponding to supercoiled (SC) DNA, was increased by the combination of CDDP with BV10 (Figs. 1A,C). The latter result indicates that the presence of BV10 decreased the formation of the well-known CDDP-DNA crosslinking that would lead to fluorescence quenching of the dye-stained DNA in gel electrophoresis imaging^27^. This implies that fewer free CDDP molecules were available to bind to DNA as CDDP molecules were involved in another reaction in the combination. These results are consistent with the hypothesis that the DET reaction between CDDP and BV10 (to be shown later) can produce reactive radicals to cause DNA DSBs.

**Figure 2:**
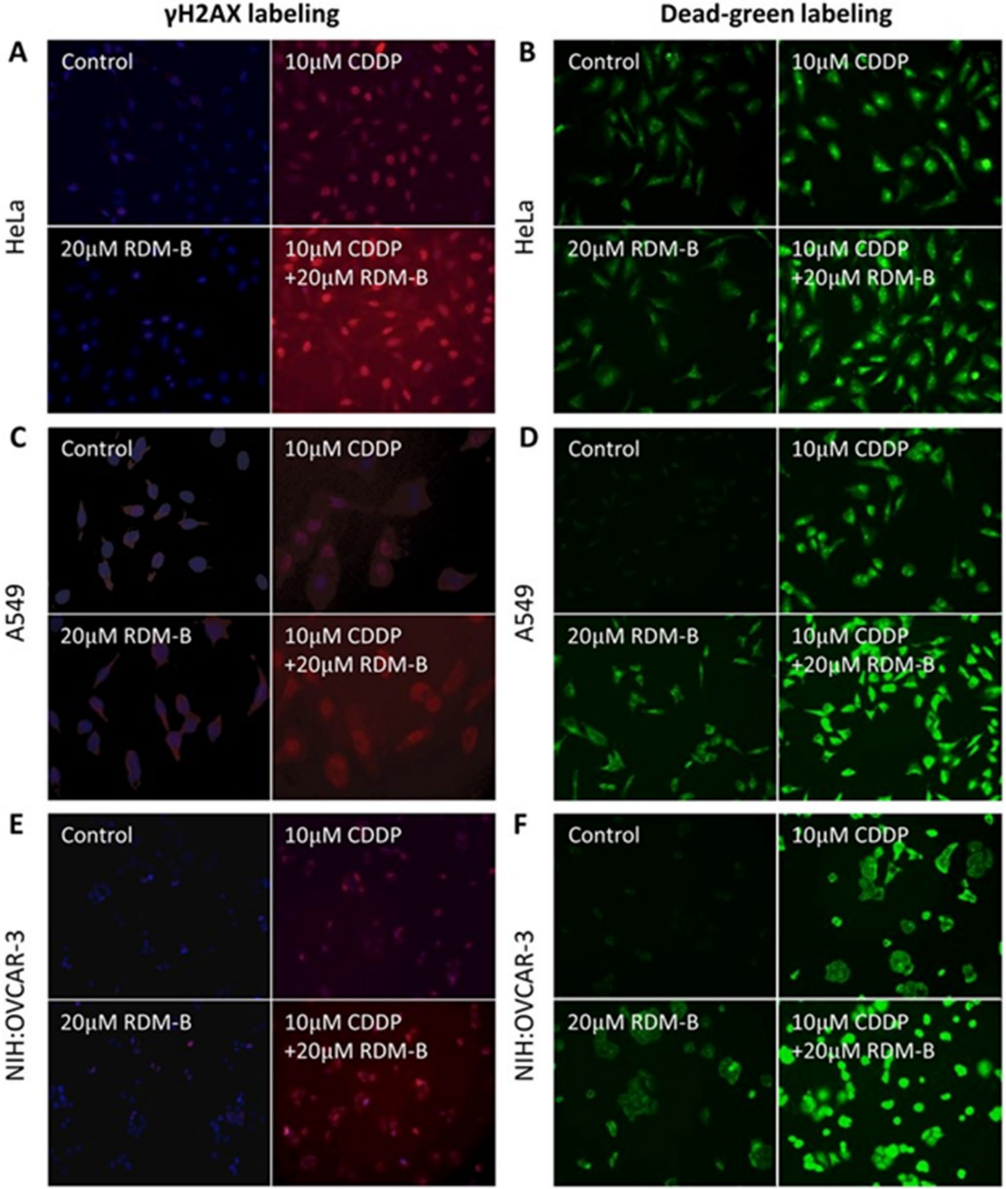
Cellular DNA DSB measurements in different cancer cell lines using γH2AX labeling. Representative pictures of γH2AX labeling are shown in (**A**), (**C**) and (**E**), and those of dead green staining are shown in (**B**), (**D**) and (**F**) in HeLa, A549 and NIH:OVACR-3 cells, respectively, with the treatments indicated.

The above gel electrophoresis results are further confirmed by measurements of cellular DNA DSBs in cervical cancer HeLa, lung cancer A549, and ovarian cancer NIH:OVCAR-3 cells (Figs. 2A,C,E, respectively). Cellular DNA with phosphorylated H2AX (the DSB repairing protein) was stained red; cells that lost membrane integrity were stained green. Representative images after 12 h treatment of CDDP with/without BV10 are shown in Figs. 2B,D,F. It could be seen that 20 μM BV10 alone induced almost no DSBs of DNA, 10 μM CDDP alone induced some DNA DSBs, and the amount of DSBs was enhanced by the combination with 20 μM BV10 by 1.7, 5.2, and 8 times respectively, on HeLa, A549, and NIH:OVCAR-3 cells. These results show that the combination of BV10 with CDDP indeed led to a significant enhancement in the formation of DNA DSBs.

### In vitro cell viability and death tests

**Figure 3:**
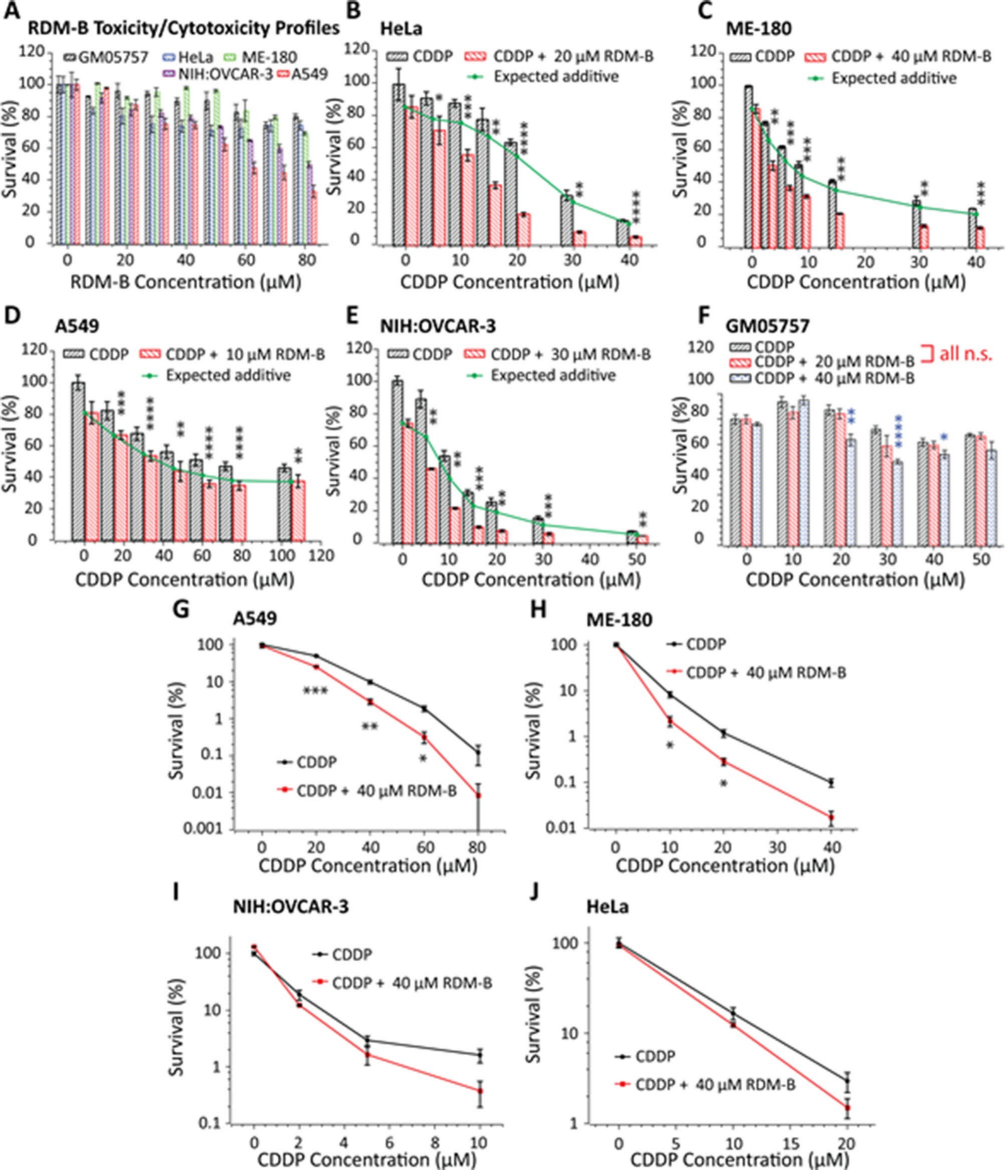
In vitro cell viability tests on various human cancer cell lines and a normal cell line. All viabilities are represented as percentages with respect to the control (untreated cells, taken as 100% survival). The MTT assay was performed to obtain RDM-B (BV10) cytotoxicity and toxicity profiles (**A**), cell-killing efficacies of CDDP and its combination with BV10 in cervical cancer HeLa cells (**B**) and ME-180 cells (**C**), lung cancer A549 cells (**D**), ovarian cancer NIH:OVCAR-3 cells (**E**), and normal cells (**F**), in which error bars represent the standard deviation of data obtained in each group. All MTT experiments were performed at 24 h post-treatment. The clonogenic assay was performed in A549 (**G**), ME-180 (**H**), NIH:OVCAR-3 (**I**), and HeLa (**J**) cells for 2 h treatment with CDDP and its combination with BV10, in which error bars represent the standard error of the mean (s.e.m.) of data obtained in each group. The *p* values reported on the graph were obtained from unpaired two-tail student *t* tests: ****, p < 0.0001; ***, p < 0.001; **, p < 0.01; *, p < 0.05; n.s., nonsignificant (p ≥ 0.05).

The results of the effect of the combination of CDDP with BV10 on in vitro cell viability and clonogenic death are shown in Fig. 3. Figure 3A shows the toxicity and cytotoxicity profiles after the 24 h BV10 treatment of various cells, including a human normal cell line (GM05757), CDDP-sensitive human cervical cancer (HeLa and ME-180), (intermediately) CDDP-resistant human lung cancer (A549), and (highly) CDDP-resistant human ovarian cancer (NIH:OVCAR-3) cells^27^. Figure 3A shows that BV10 itself at low concentrations (≤ 40 μM) induced only slightly killings of both normal and cancer cells with viabilities of 80–95%. Figures 3B–F show the effects of the combination of CDDP and BV10 in treating various cancer cells and normal cells. For all the 4 cancer cell lines, the observed cell viabilities by the combination treatment were lower than the ‘expected additive’ calculated by the fractional effect method^30^. This indicates that CDDP and BV10 killed these cancer cells in a synergistic manner. Quantitatively, the IC_50_ of CDDP alone (the concentration required to kill 50% of untreated cells) for HeLa cells was determined to be 25 ± 2 μM, which was reduced to 11 ± 2 μM with the combination with BV10. Similarly, the IC_50_ for ME-180 cells was reduced from 10 ± 1 μM for CDDP alone to 3 ± 1 μM for the combination. Interestingly, this combination was found to be less effective in killing normal cells (Fig. 3F). Figures 3G–J show the cell survival results from the clonogenic assay. It is notable that for the CDDP-resistant lung cancer A549 cell line, the cell survival was dramatically decreased by more than one order of magnitude for the combination with BV10, compared to that by CDDP alone. Strong synergy was also observed on the other three cancer cell lines. These results show that the combination with BV10 strongly enhanced the cell-killing efficacy of CDDP in cancer cells in a synergistic manner.

### In vitro apoptosis measurements

**Figure 4:**
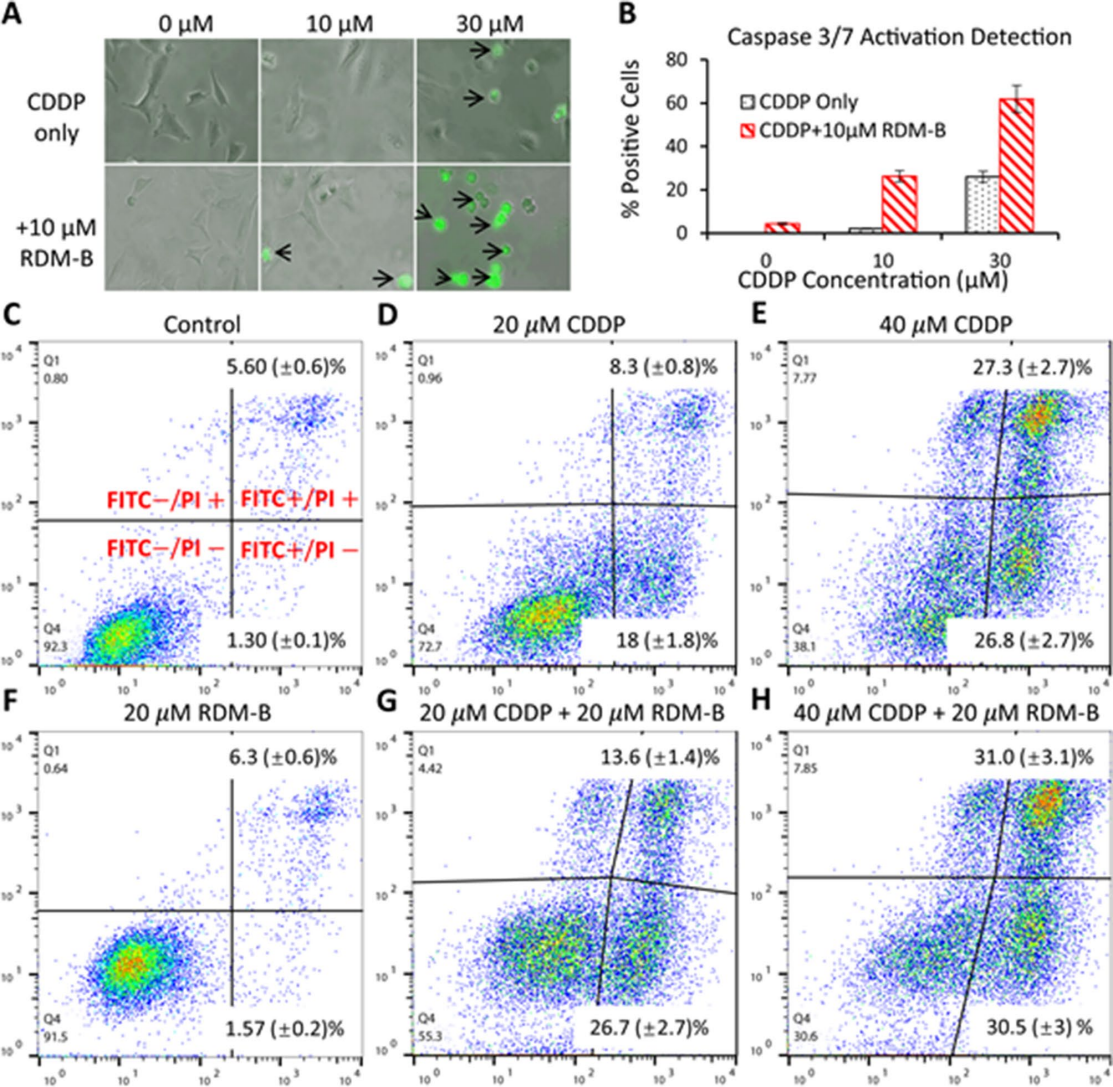
In vitro apoptosis detection in cervical cancer HeLa cells. (**A**) Representative pictures (black arrows indicating morphological changes) of HeLa cells treated with 10 μM and 30 μM CDDP with/without the combination of 10 μM RDM-B (BV10) for 12 h and then incubated with the CellEvent Caspase-3/7 Green Detection Reagent and analyzed using fluorescence microscopy. (**B**) Percentages of activated caspases 3/7. (**C**)–(**H**) Early/late apoptosis measurements of HeLa cells treated with 20 μM and 40 μM CDDP with/without the combination of 20 μM BV10 for 18 h using an Annexin V-FITC Apoptosis Detection Kit, where the cells were double stained with Annexin-V-FITC and PI. Quantitative analyses of the cell images in A and flow cytometry data in (**C**)–(**H**) were performed using an ImageJ software (https://imagej.nih.gov/ij/) and a FlowJo software (https://www.flowjo.com/solutions/flowjo), respectively.

Figure 4A shows representative images of HeLa cells treated by CDDP with/without the combination with BV10 for 12 h, where apoptotic cells were stained green. Quantitative results are shown in Fig. 4B, which shows that 10 μM BV10 induced almost no apoptosis. When it was combined with 30 $\mathrm{\mu M}$ CDDP, an enhancement by 2.4 times of apoptotic cells was observed, compared with the CDDP only treatment. Apart from caspase activation, apoptosis was also characterized by morphologic changes (black arrows in Fig. 4A), including the loss of cell membrane asymmetry and attachment, blebbing, cytoplasm and nucleus condensation, and DNA fragmentation.

Early apoptosis is characterized by membrane phospholipid phosphatidylserine (PS) translocation from the inner leaflet to the outer, and late apoptosis is characterized by damaged cell membrane. Therefore, FITC conjugated Annexin V (with high affinity for PS) and a vital dye propidium iodide (PI) were used to simultaneously detect early and late apoptosis, respectively. Healthy cells were stained FITC − /PI − , early apoptotic cells were stained FITC + /PI − , and late apoptotic/dead cells were stained FITC + /PI + . The results for the HeLa cells treated with 20 μM and 40 μM CDDP with/without the combination of 20 μM BV10 for 18 h are shown in Figs. 4C–H. As shown in Fig. 4F, 20 μM BV10 alone induced no significant early/late apoptosis with a decrease of only as low as 0.8% in the population of healthy cells, compared with the control (Fig. 4C). However, when 20 μM BV10 was combined with CDDP, a dramatic increase in the populations of early/late-apoptotic cells was observed (Figs. 4D,E,G,H). At 20 μM CDDP, the combination with 20 μM BV10 increased the damaged cell population from 27.3 to 44.7% (Figs. 4D,G). Also, by comparing the cells treated with the CDDP-BV10 combination and those treated by CDDP only, it is notable that the cells moved collectively upwards to the PI + direction. These cells were washed three times with PBS before staining was performed. Therefore, this movement should be caused by residual BV10 locating inside the cells, which has overlapped excitation and fluorescence spectra with the fluorochrome PI. This observation provides evidence that BV10 can enter the cell during incubation, rendering it possible for the reaction of CDDP and BV10 to occur inside the cells. The results in Fig. 4 show that the enhanced cell-killing efficacy of the combination of CDDP and BV10 was caused by inducing more apoptosis.

### In vivo anti-cancer efficacy tests in xenograft mouse models of cancers

**Figure 5:**
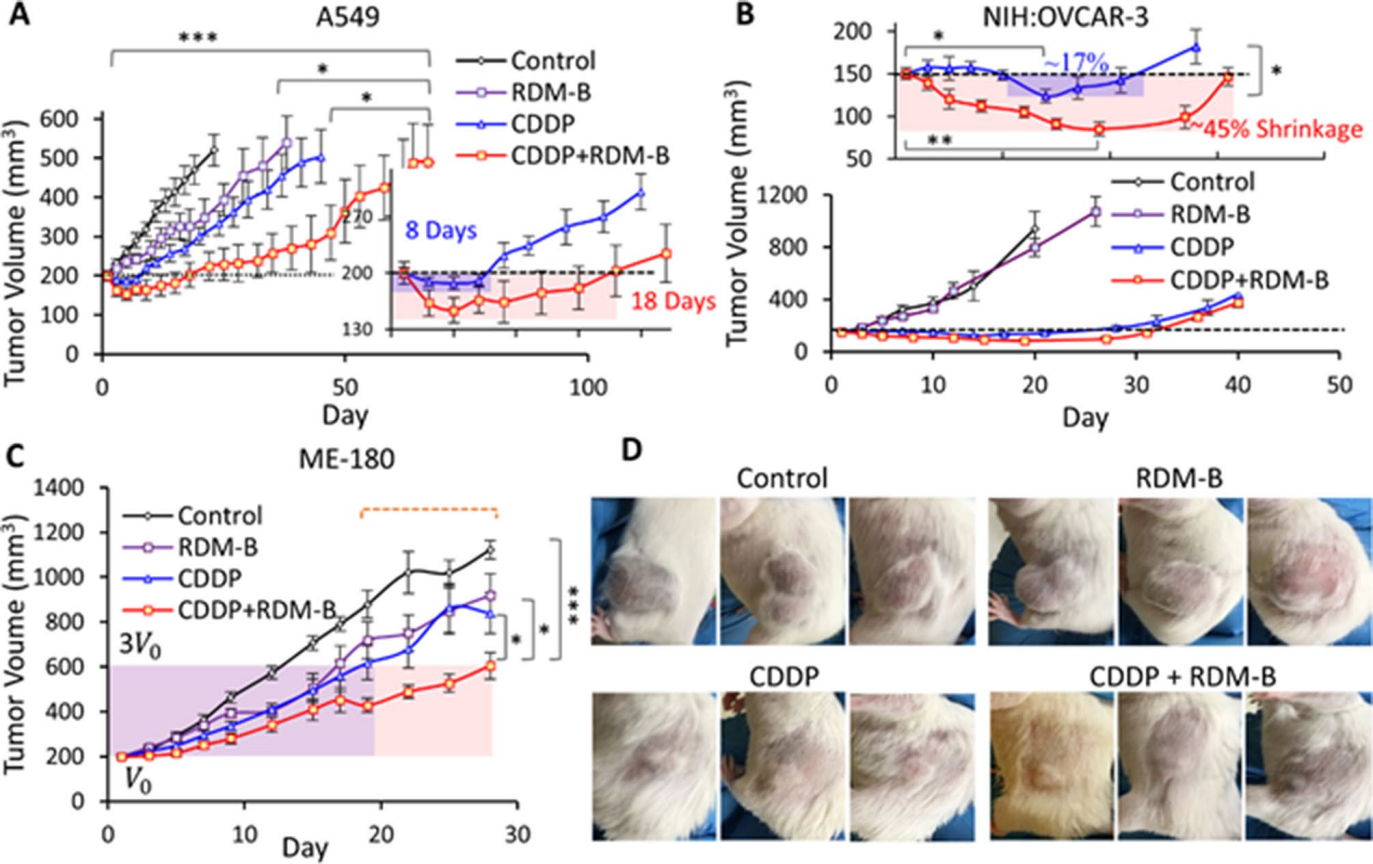
Mouse xenograft models of human lung (A549) cancer, human cervical (ME-180) cancer, and human ovarian (NIH:OVCAR-3) cancer treated by CDDP or RDM-B (BV10) alone and their combination. (**A**–**C**) Tumor growth curves for the A549, NIH:OVCAR-3, and ME-180 models, respectively, where error bars represent the standard error of the mean (s.e.m.). (**D**) Representative pictures of mice bearing ovarian tumors. The statistical analysis results indicated in (**A**)–(**C**) were obtained from either two-way ANOVA (multiple groups in **A** and **C**) or unpaired two-tail student t tests (two groups in **B**): ***, p < 0.001; **, p < 0.01; *, p < 0.05; n.s., nonsignificant (p ≥ 0.05). Statistical analyses were performed with GraphPad Prism 9.0.0 (https://www.graphpad.com/scientific-software/prism/) and Microsoft Excel.

After observing the enhanced cell-killing efficacy of the combination of CDDP and BV10 in various cancer cell lines and studying cell death processes, we further investigated the anti-cancer effect of this combination in the xenograft mouse models of lung, ovarian and cervical cancers. The typical dose of CDDP in xenograft mouse models is 2.0–7.5 mg/kg per treatment^31,32^. For our present experiments, CDDP was administered at 2.0 mg/kg × 3 (3 treatments) in the cisplatin-sensitive cervical cancer ME-180 model, 2.5 mg/kg × 3 in the (intermediately) cisplatin-resistant lung cancer A549 model, and 2.5 mg/kg × 4 in the (highly) cisplatin-resistant ovarian cancer NIH:OVCAR-3 model; RDM-B (BV10) (alone or in the combination) was administered at 8.0 mg/kg per treatment. The treatments were given every other day. For the three xenograft tumor models, tumor growth curves are shown in Fig. 5, whereas measured volumes of tumours at day 1 (pre-treatment), 5, 10, and 20 of the treatments are given in Table 1.

**Table 1:** Measured volumes of tumours of lung (A549)/ovary (NIH:OVCAR-3)/cervix (ME-180) at day 1, 5, 10, and 20 of the treatments by the vehicle (control), 8 mg/kg RDM-B (BV10) only, 2.0/2.5 mg/kg CDDP only, and 2.0/2.5 mg/kg CDDP + 8 mg/kg RDM-B (BV10). All tumour volumes were normalized to the Day 1 value (200 mm^3^ for the lung and cervix models, 150 mm^3^ for the ovary model).

Treatment	Day 1 (mm^3^) Lung/ovary/cervix	Day 5 (mm^3^) Lung/ovary/cervix	~ Day 10 (mm^3^) Lung/ovary/cervix	~ Day 20 (mm^3^) Lung/ovary/cervix
Control	200/150/200	260/248/293	318/374/463	444/941/878
RDM-B	200/150/200	236/240/283	265/334/393	326/799/718
CDDP	200/150/200	187/156/249	222/149/338	270/142/617
CDDP + RDM-B	200/150/200	154/120/215	164/105/280	203/85/428

As seen from Fig. 5A and Table 1, the 8 mg/kg $\times$ 3 BV10 treatment only weakly inhibited the tumor growth in the lung cancer model, whereas the 2.5 mg/kg $\times$ 3 CDDP treatment induced mild tumor shrinkage (from the volume of 200 mm^3^ to the lowest of ~ 187 mm^3^) during the treatment period (day 1–5) and it took 3 days only to regrow back to the pre-treatment volume. In comparison, the (2.5 mg/kg CDDP + 8 mg/kg BV10) × 3 combination treatment induced a much more significant tumor shrinkage (to the lowest volume of ~ 154 mm^3^). Moreover, it is worthwhile noting that the combination group largely prolonged the tumor shrinkage period from 8 to 18 days, compared to the CDDP alone group.

Tumor growth measurements of the other two xenograft models also showed a dramatic difference between the CDDP alone and the combination groups. For the highly CDDP-resistant ovarian cancer model, as shown in Fig. 5B, the tumor-size curves of control and BV10 alone groups increased at a similar speed, and no significant difference was observed. The averaged tumor volume in the CDDP alone group decreased in the period of day 10 to day 17, and the tumor growth inhibition after the treatment was significant (~ 15 days before apparent tumor regrowth). In contrast, the treatment of (2.5 mg/kg CDDP $+$ 8 mg/kg BV10) × 4 induced tumor shrinkage even during the initial treatment (at days 1–7), while the 2.5 mg/kg CDDP × 4 group induced no tumor shrinkage in this same period. It is even more remarkable that for the combination treatment group, the tumor volume kept decreasing to the lowest volume of 85 mm^3^ on day 18 with ~ 45% tumor shrinkage and it took approximately 25 more days to re-grow back to the pre-treatment volume of 150 mm^3^. As also seen from the pictures (taken on day 22) in Fig. 5D, BV10 alone had almost no effect on the tumor growth; when BV10 was combined with CDDP, a drastically different result was observed: the tumor growth inhibition was significantly enhanced.

For the CDDP-sensitive cervical cancer ME-180 model (Fig. 5C), the ME-180 tumor was more sensitive to the 8 mg/kg BV10 treatment, compared to the other two models of A549 and NIH:OVCAR-3. Tumor growth was inhibited by both single agent treatment of BV10 or CDDP, with a slightly better result observed in the CDDP group. The combination of CDDP and BV10 led to more significant inhibition of the tumor growth, though no significant tumor shrinkage was observed for any of the BV10/CDDP alone and BV10-CDDP combination treatments in this cancer model. Specifically, tumor-volume-tripling time was 12 days for the control group, 17 days for the BV10 group, 19 days for the CDDP group, and 28 days for the combination group.

Figure S1 in Supplementary Data tabulates acute toxicity test results for the CDDP-resistant ovarian cancer NIH:OVCAR-3 model, since mice in this model received the highest accumulated doses of CDDP (10 mg/kg) and BV10 (32 mg/kg). These tests provided information on hepatotoxicity represented by serum alkaline phosphatase (ALP), alanine aminotransferase (ALT), and total bilirubin (TBIL) levels, and nephrotoxicity represented by serum urea and creatinine levels. As can be seen in Figure S1, none of the given treatments induced significant toxicity to either the kidney or the liver under the used dosages, and no significant electrolyte imbalance was observed in the treated mice. All the levels were in the normal ranges, which were also given as references in Figure S1. Comparing the in vivo apoptosis induced by different treatment groups (Figure S2), we found that cisplatin alone caused much stronger gut toxicity than BV10 alone, while the combination of BV10 to CDDP caused no significant additional gut damage.

In summary, the above in vivo results have clearly demonstrated that the combination of CDDP and BV10 strongly potentiate the anti-tumor effect of CDDP in treating cancers with various degrees of CDDP resistance. More remarkably, the combination treatment in the two CDDP-resistant models (A549 and NIH:OVCAR-3) dramatically increased the duration of tumor shrinkage. In addition, these enhancements in anti-tumor effects were obtained with little sacrifice in the overall health conditions of the treated mice, as shown in the results of toxicity tests.

### Femtosecond time-resolved laser spectroscopy (fs-TRLS) measurements

**Figure 6:**
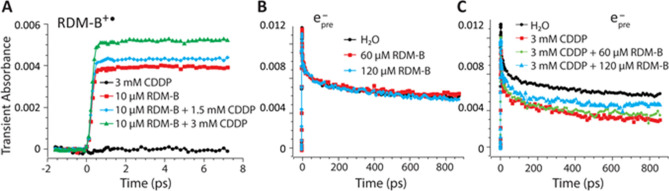
Femtosecond time-resolved (pump-probe) laser spectroscopic observations of the dissociative electron transfer (DET) reaction between cisplatin (CDDP) and Rhodamine-B (BV10) in aqueous solutions. (**A**) Transient absorption measurements of BV10 cation radical (RDM-B^+•^) in 10 μM BV10 only, 3 mM CDDP only, and the mixtures of 10 μM BV10 and 1.5 mM or 3 mM CDDP. (**B**,**C**) Transient absorption measurements of prehydrated electrons (e_pre_^−^) in pure water, 60 μM and 120 μM BV10 only solutions, and in pure water, 3 mM CDDP only and the mixtures of 3 mM CDDP with 60 μM or 120 μM BV10.

To demonstrate the DET mechanism of the combination, we made real-time measurements of the reaction between CDDP and BV10 (RDM-B) expressed in reaction (2). The photo-degradation of BV10 has been well studied previously^33,34^, and the initial step involves the photo-excitation and one electron oxidization of BV10 by electron-transfer to oxygen^33^. The resultant cation radical (BV10^+•^) was observed to have an absorption peak at 490 nm^34^. Therefore, by probing the real-time formation of the species BV10^+•^ with/without the presence of CDDP, we could obtain information on the reaction between CDDP and BV10. A standard femtosecond transient absorption measurement was carried out on BV10 water solutions with/without the presence of CDDP. The pump pulse was chosen at 553 nm (0.2 μJ/pulse) that is the absorption peak of BV10, and the probe pulse was chosen at 490 nm to monitor the real-time formation of the radical BV10^+•^. Since the DET reaction between CDDP and a weakly-bound electron is an ultra-fast process that occurs within 1 picosecond (ps) ^20,21^, we measured the transient absorption for only ~ 8 ps after the zero time point. As can be seen in Fig. 6A, the photo-excitation by 553 nm pulses in the 10 μM BV10 only aqueous solution gave rise to the formation of BV10^+•^; as 1.5 mM and 3 mM of CDDP was added to the 10 μM BV10 solution, the yield of BV10^+•^ was increased in a CDDP-concentration-dependent manner. These results show that there exists an electron transfer (ET) reaction between CDDP and (photoexcited) BV10; the presence of CDDP accelerated the photo-degradation of BV10, resulting in the increased yield of BV10^+•^. It suggests that in the mixed solution of both BV10 and CDDP, BV10 donated electrons not only to oxygen but also to CDDP.

To further study the nature of the above-observed electron-transfer reaction, we conducted the fs-TRLS transient absorption measurements to monitor the competing reactions with CDDP of ground-state (un-photoexcited) BV10 versus weakly-bound prehydrated electrons (e_pre_^−^). In this experiment, the pump pulse was chosen at 266 nm (0.2 μJ/pulse), at which the two-photon absorption by water molecules generates e_pre_^−^^24^. e_pre_^−^ has absorption in the near-infrared region, so the probe pulse was chosen at 800 nm to conveniently monitor the formation of e_pre_^−^^24^. Figure 6B shows the transient absorption measurements of e_pre_^−^ in pure water, 60 and 120 μM BV10 aqueous solutions, which shows that upon the pump pulse excitation, e_pre_^−^ was generated, and when BV10 was added, the concentration of e_pre_^−^ was not affected as no light was absorbed by BV10 at this pump excitation (i.e., no photo-excitation of BV10 occurred). Then we conducted the transient absorption measurements of e_pre_^−^ in the solutions of 3 mM CDDP only, and 3 mM CDDP plus 60 μM or 120 μM BV10. Figure 6C clearly shows that the presence of CDDP significantly decreased the concentration of e_pre_^−^ due to the highly effective DET reaction^20^. It is interesting to observe that when compared with the 3 mM CDDP only solution, the addition of BV10 increased the concentration of e_pre_^−^ in an BV10-concentration-dependent manner. This result indicates that with the presence of ground-state (un-photoexcited) BV10, more e_pre_^−^ survived in this oxidizing system (with CDDP), implying that the neutral ground-state BV10, by donating its electrons, competed with e_pre_^−^, to react with CDDP. This competing reaction led to the observation that the yield of the surviving e_pre_^−^ produced from the radiolysis of water increased in the combination solutions. These results provide strong evidence that BV10 reacts with CDDP, and this reaction is an electron transfer reaction. The competing DET reactions with CDDP can be expressed as the following:

3a $${\mathrm{e}}_{\mathrm{pre}}^{-}+\mathrm{Pt}{({\mathrm{NH}}_{3})}_{2}{\mathrm{Cl}}_{2}\stackrel{ }{\to }{[\mathrm{Pt}{({\mathrm{NH}}_{3})}_{2}{\mathrm{Cl}}_{2}]}^{*-}\stackrel{ }{\to }\mathrm{Pt}{({\mathrm{NH}}_{3})}_{2}{\mathrm{Cl}}^{\bullet}+{\mathrm{Cl}}^{-}$$

3b $$\mathrm{RDM}-\mathrm{B}+\mathrm{Pt}{({\mathrm{NH}}_{3})}_{2}{\mathrm{Cl}}_{2}\stackrel{ }{\to }{\mathrm{RDM}-\mathrm{B}}^{+\bullet}+{\mathrm{Cl}}^{-}+\mathrm{Pt}{({\mathrm{NH}}_{3})}_{2}{\mathrm{Cl}}^{\bullet}$$

With the combination of BV10 (RDM-B) with CDDP, $\mathrm{Pt}{({\mathrm{NH}}_{3})}_{2}{\mathrm{Cl}}^{\bullet}$ and $\mathrm{Pt}{{({\mathrm{NH}}_{3})}_{2}}^{\bullet}$ radicals are generated to induce DNA strand breaks and then cause cell death, as observed in Figs. 1–4.

## Discussion

This study demonstrates that the DET-based combination regimen of CDDP can effectively enhance the anti-cancer activity of CDDP for the treatment of various cancers with various degree of CDDP resistance. Our in vitro results show that the combination with BV10 dramatically potentiated the cell-killing efficacy of CDDP in various cancer cells, by inducing more DNA double-strand breaks and apoptosis. Furthermore, the combination of BV10 with CDDP showed selectivity in enhancing the efficacy of CDDP between cancer cells and normal cells. More remarkably, our in vivo xenograft mouse models show that the anti-tumor effect of CDDP was significantly enhanced with the combination with BV10, in contrast to the combination of cisplatin with TMPD that showed effective in vitro but no in vivo^27^. Notably, the combination induced more significant tumor shrinkage and the shrinkage duration was significantly prolonged for the two CDDP-resistant tumor models (A549 and NIH:OVCAR-3). Also, the acute toxicity tests showed that the combination of CDDP and BV10 induced no additional toxic side effects. Furthermore, our real-time femtosecond laser spectroscopic measurements confirmed the electron transfer reaction between CDDP and BV10. By probing the radical cation BV10^+•^ formed in aqueous BV10 solution, it was found that the addition of CDDP significantly increased the yield of BV10^+•^, which indicates that CDDP captured electrons from BV10. Furthermore, by probing the competing reactions of BV10 versus e_pre_^−^ produced from 2-photon UV absorption of water with CDDP, it was observed that the addition of BV10 increased the concentration of e_pre_^−^ compared to the pure CDDP solution. The latter result indicates that BV10 competed with e_pre_^−^ to react with CDDP. Once an electron is transferred to CDDP, the latter will rapidly dissociate to form a radical causing biological effects^20–23,27^. Therefore, our real-time transient absorption measurements confirm the DET reaction between CDDP and BV10. Interestingly, other researchers have previously synthesized a Pt(II)-activatable Rhodamine-B-based fluorogenic probe for imaging intracellular Pt species after cisplatin exposure^35^.

The observation of the combination of BV10 with CDDP selectively killing cancer cells and inducing no or minimal additional toxic side effects can be well explained by the observed DET mechanism. Based on the reductive DNA damaging mechanism^25,26^, it can be deduced that cancer cells tend to have a more reduced (electron-rich) intra-cellular environment than normal cells^36,37^. Moreover, the most marked feature in the microenvironment of tumors is hypoxia, which has multiple consequences for tumor progression and treatment outcome. This means that an electron-donating PM_x_ in reaction (2) will become oxidized in (oxic) normal cells while it keeps its reducing (electron-donating) capacity in cancer cells or hypoxic tumor, that is, the DET reaction between CDDP and the PM_x_ will be preferentially effective in cancer cells or in the hypoxic tumor microenvironment where there is low-level O_2_ to oxidize the PM_x_. This thus provides a method of targeting cancer cells while inducing little systemic toxicity.

The observed results indicate that by combining CDDP with BV10, the resistance of cancer cells to CDDP can be circumvented significantly and the required dose of CDDP can be reduced to achieve the same chemotherapeutic effect of CDDP. It means that the therapeutic window of CDDP can be broadened and the toxic side effects associated with the heavy metal Pt can be reduced^8,9^. This proposed combination also allows a better chemotherapeutic effect at the same maximum tolerated dose (MTD) of CDDP. CDDP-induced hepatotoxicity is rare when standard doses are administered; however, there are many circumstances where higher doses or repeated low-dose treatments are required for effective tumor suppression. In these cases, hepatotoxicity is not negligible and has become a dose-limiting factor in the clinic. Our results indicated that the newly found combination could enhance the anti-tumor effect without inducing significant additional hepatotoxicity; therefore, the problem of requiring high-dose CDDP treatments for some cancers may be overcome by the proposed combination.

A desired DET-based combination is CDDP plus a non-toxic electron donating agent. Therefore, the concern on the safety using BV10 should be addressed. Despite its wide applications in industries including paints, papers, leathers, etc., the use of BV10 in food and cosmetics has been prohibited due to the concern of its potential toxicity. However, the proposed use of BV10 as a safe drug requires to be further investigated. First, the reported intraperitoneal LD_50_ of BV10 is 144 mg/kg in mice, while the results of our trials have indicated that a dose of as low as 8 mg/kg was sufficient to generate a significant enhancement in the therapeutic efficacy of CDDP, resulting remarkably in tumor shrinkage. It is worthwhile to note that in our in vivo studies, the used dose of CDDP 2.5 mg/kg is 38% of its reported intraperitoneal LD_50_ dose in mice (6.6 mg/kg) and that of BV10 is only 5.5% of its IP LD_50_ value. It follows that the effective dose of BV10 in this combination is far below the dose that could induce significant acute toxicity, as observed in the present study. Second, some studies suggested a potential carcinogenic effect of BV10 when it was administered with diet for a long period of 22–29 months^38^, which should be a long-term accumulated effect from persistent exposure to the compound. While in our studies, as well as in the clinic, chemotherapy is usually given in a short period with a much longer recovery period between treatments. According to FDA’s cisplatin administration guideline, “PLATINOL should not be given more frequently than once every 3 to 4 weeks”. Finally, another critical rationale for the proposed combination is that CDDP itself is a much stronger carcinogenic agent than BV10, and therefore the reduction of the CDDP dose by the introduction of the latter should be beneficial to the patients receiving the treatment. Our acute toxicity analysis has also shown that the administration of BV10 at the used dose introduced no significant nephrotoxicity and hepatotoxicity in mice. Therefore, the proposed combination of CDDP at 2.0–2.5 mg/kg with BV10 at 8.0 mg/kg is not only effective but safe under the indicated administration schedule.

## Methods

### Chemicals and reagents

Rhodamine-B (BV10) and cis-Diammineplatinum(II) dichloride (cisplatin, CDDP) were purchased from Sigma-Aldrich without further purification. A 3 mM stock solution of CDDP and a 24 mM stock solution of BV10 were both prepared in ultrapure water (resistivity > 18.2 M $\Omega$/cm, TOC < 1 ppm) obtained from a Barnstead Nanopure water system.

### Fs-TRLS transient absorption measurements

Ultrafast experiments were conducted through a pump-probe transient absorption setup^24–26^. A Ti:sapphire laser system (Spectra-Physics) produced 800 nm laser pulses with a pulse duration of 100–120 fs and a repetition rate of 500 Hz. Two optical parametric amplifiers (OPA) were applied to produce pump and probe pulses with desired wavelengths from visible to IR. The polarization of the pump pulse with respect to the probe pulse was set to be at 54.7 $^\circ$ (magic angle) to avoid polarization anisotropy. For the experiment studying the DET reaction of CDDP with photo-stimulated BV10, pump and probe pulse wavelengths were chosen at 553 nm and 490 nm, respectively, and the pump energy was 0.2 μJ. For the experiment studying the DET reactions of CDDP with prehydrated electrons and ground-state BV10, pump and probe pulse wavelengths were chosen at 266 nm and 800 nm, respectively. To void product accumulation, all samples were measured in a 5 mm quartz cell at room temperature and stirred during measurements.

### Cell lines and culture conditions

Human cervical cancer HeLa cell line (ATCC#: CCL-2), human cervical cancer ME-180 cell line (ATCC#: HTB-33), human non-small-cell lung cancer A549 cell line (ATCC#: CCL-185), and human ovarian cancer NIH:OVCAR-3 (ATCC# HTB-161) cell line were obtained from the American Type Culture Collection (ATCC, Manassas, VA, USA). Human skin diploid fibroblast GM05757 normal cell line was obtained from the Coriell Cell Repository directly. The culture conditions of these cells were reported previously^27–29^. Particularly, the NIH:OVCAR-3 cell line has been established as a model system for studies of the resistance of CDDP and other chemotherapy drugs^39,40^.

### Agarose gel electrophoresis

Plasmid DNA (Pgem 3Af(-), 3197kbp) was extracted from *Escherichia Coli* JM109 and purified using the GeneJET Plasmid Miniprep Kit (Thermo Scientific). The experimental details were given previously^20,26,27^.

### In vitro DNA double-strand breaks (DSBs) measurements

The HCS DNA Damage Kit was purchased from Invitrogen. The kit was mainly used to detect and quantitate in vitro genotoxicity, specifically the form DNA DSBs. This kit was also used to detect in vitro cytotoxicity by Image-iT Dead Green viability staining. This kit also allows Hoechst 33342 nuclear staining in both dead and live cells. The experimental details were given previously^27,28^.

### Cell viability assays

In vitro cell viabilities were determined by the MTT (3-(4,5-dimethylthiazol-2-yl)-2,5-diphenyltetrazolium bromide) assay and the clonogenic assay with the details described previously^27–29^.

### Caspase 3/7 activation detection

Apoptosis detection was performed by using the CellEvent Caspase-3/7 Green Detection Reagent (Invitrogen), as described previously^27,28^**.**

### Early/late apoptosis discrimination

The Annexin V-FITC Apoptosis Detection Kit was purchased from Sigma-Aldrich. Fluorescence detection of FITC and PI was performed by flow cytometry. 5 $\times {10}^{5}$ cells were seeded into T25 flasks, and incubated for overnight. Treatment was given when the cells reached 50% confluency. After the treatment, cells were collected, centrifuged, washed, resuspended, and then labeled by Annexin V-FITC conjugate and PI solutions, following the protocol from the manufacturer. After 10 min incubation in dark, cells were analyzed by a flow cytometer (BD FACSAria Fusion) for fluorescence detection of single cells. In each experiment, one unstained sample (with no treatment and no staining) and two single-stained positive samples (FITC only and PI only) were prepared to help with gating. Quantitative analysis was performed by the software FlowJo (https://www.flowjo.com/solutions/flowjo).

### Mice and xenograft cancer models

Female SCID mice aged at 6–8 weeks (Charles River) were used. Mouse xenograft models of lung cancer (A549), ovarian cancer (NIH:OVCAR-3), and cervical cancer (ME-180) were developed, as described previously^28,29^. Briefly, 100 $\mathrm{\mu L}$ culture solutions containing 5 $\times {10}^{6}$ A549 cells, 6 $\times {10}^{6}$ NIH:OVCAR-3 cells and 2 $\times {10}^{6}$ ME-180 cells were injected into the left flank of mice through subcutaneous injection to establish the xenograft mouse models of A549 lung, NIH:OVCAR-3 ovarian, and ME-180 cervical cancers, respectively.

### Chemotherapy treatment in mice^28^

Mice with tumor volumes reaching 200 mm^3^ (A549 and ME-180) or 150 mm^3^ (NIH:OVCAR-3) were randomly allocated into 4 groups: control, BV10, CDDP, and CDDP + BV10, with 5 mice/group. Mice in the control received saline injections to eliminate the deviation caused by the injections itself. All injections were performed by intraperitoneal (IP) injections. Cisplatin and BV10 solutions were separately prepared and injected with two syringes. The two extra mice from each group in the A549 and NIH:OVCAR-3 trials were euthanized 24 h after the last treatment for acute toxicity analysis.

### Tumor volume measurements^28,29^

Mice were weighed, and tumors were measured three times a week right after treatments, and twice a week after two weeks from day 1. The tumor volume was calculated by the well-accepted formula: $\mathrm{V}=\mathrm{L}\times \frac{{\mathrm{W}}^{2}}{2}$, where L and W are the measured lengths of the longest axis and the axis perpendicular to it, respectively. When multiple tumors developed, the tumor size was calculated as the summation of individual small tumors.

### In vivo acute toxicity analysis

Two mice from each group were euthanized 24 h after the last treatment for acute toxicity study^28,29^. Their blood samples were collected through a terminal cardiac puncture in mice. Serum samples were then analyzed to study hepatotoxicity and nephrotoxicity. Nephrotoxicity also causes electrolyte disturbances. Thus, serum electrolytes’ levels were also measured.

### In vivo apoptosis detection in the gut

Gut tissue samples were collected from tumor-bearing (NIH:OVCAR-3) mice 24 h after the last treatment and were stored in formalin. After being sectioned and fixed on glass slides, the standard TUNEL (terminal deoxynucleotidyl transferase (TdT) dUTP nick end labeling) assay was performed using the DeadEnd Colorimetric TUNEL System (Promega). The processed slides were then observed under the microscope and pictures were taken with a Nikon Cloopix 8400 camera. Apoptotic cells were stained dark brown and non-apoptotic cells were stained blue.

### Animal experiments guidelines and humane endpoints

All animal experiments were carried out in compliance with the guidelines of the University of Waterloo Animal Care Committee with an approved Animal Utilization Project Protocol (AUPP #30062) and with the ARRIVE guidelines. Humane endpoints were reached if any of the following was observed: (1) body weight loss > 20% from the first day of treatment; (2) diameter of the longest axis of the tumor > 17 mm; (3) body condition score (BCS) < 2; (4) deep tumor ulceration.

### Statistical analysis

The data are expressed as mean value ± SD (standard deviation) or ± s.e.m. (standard error of the mean), as indicated in figure captions. In each cell viability experiment, at least three replicates were set for any treatment group. All quantitative experiments were conducted with at least two independent experiments. For all cell culture survival and in vivo tumor growth experiments, the unpaired (unequal variance) two-tail student *t* test was used to compare two groups and two-way ANOVA was performed to compare multiple groups. *P* values less than 0.05 were considered significant and are indicated in the figures as: ****, *p* < 0.0001; ***, *p* < 0.001; **, *p* < 0.01; *, *p* < 0.05; n.s., nonsignificant (*p* ≥ 0.05). Analyses were performed using Microsoft Excel and GraphPad Prism 9.0.0 (https://www.graphpad.com/scientific-software/prism/).

## Supplementary information

Supplementary information.

## Data Availability

The datasets generated during and/or analysed during the current study are available from the corresponding author on reasonable request.
