# Supplementary material for: New combination chemotherapy of cisplatin with an electron-donating compound for treatment of multiple cancers
Source: Sci Rep. 2021 Jan 12;11:788. doi: 10.1038/s41598-020-80876-z (PMC7804005; doi:10.1038/s41598-020-80876-z)
Supplement: Supplementary file 1 — Supplementary information. [file 41598_2020_80876_MOESM1_ESM.pdf]

Supplementary Data for

New combination chemotherapy of cisplatin with an electron-donating compound for treatment of multiple cancers

Qinrong Zhang[1] and Qing-Bin Lu[1,2,*]

[1] Department of Physics and Astronomy, Faculty of Science, University of Waterloo, 200 University Avenue West, Waterloo, Ontario, Canada N2L 3G1

[2] Department of Biology and Chemistry, Faculty of Science, University of Waterloo, 200 University Avenue West, Waterloo, Ontario, Canada N2L 3G1

* qblu@uwaterloo.ca

This file includes:

Figure S1. A: Mouse body weight variations in the xenograft models of lung (A549) and ovarian (NIH:OVCAR-3) cancers for the control group and the groups treated by CDDP without and with the combination with RDM-B (BV10). It was observed that CDDP alone induced significant body weight decreases, whereas RDM-B (BV10) alone induced no body weight decreases and its combination with CDDP resulted in no additional body weight changes (P>0.05). B: Acute toxicity analysis in mice: the hepatotoxicity (ALT, ALP, TBIL), nephrotoxicity (blood urea, creatinine), and electrolytes (Na, K, Cl) were measured from the blood samples collected from the mice at 24 hours after the last treatment of the NIH:OVCAR-3 model, where ALP = lkaline phosphatase, ALT = alanine aminotransferase, and TBIL = total bilirubin.

Figure S2. Representative TUNEL pictures of gut sections from NIH:OVCAR-3 tumor-bearing mice receiving different treatments.

A. Body Weight Measurements

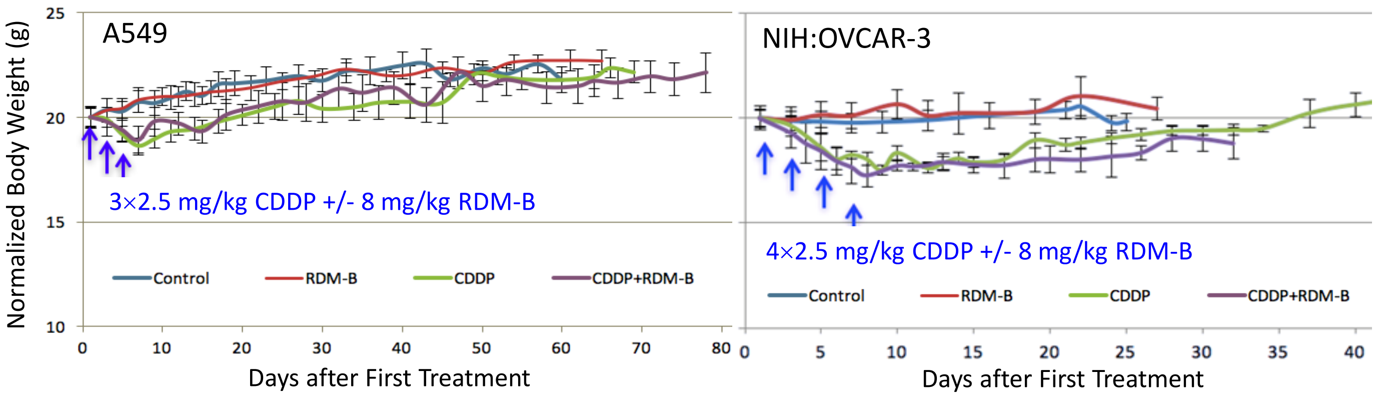

B: Acute Toxicity Test Results of NIH:OVCAR-3 Model					
Hepatotoxicity					
	Normal Range	Control	RDM-B	CDDP	CDDP+RDM-B
ALP	35-96 U/L	61 (+/-3) U/L	60.5 (+/-2.5) U/L	62.5 (+/-0.5) U/L	62 (+/-12) U/L
ALT	20-120 U/L	30 (+/-4) U/L	25 (+/-1.5) U/L	31.5 (+/-6) U/L	52.5 (+/-2) U/L
TBIL	0-15.39 μM	1.5 (+/-0.7) μM	2 (+/-0) μM	1 (+/-0) μM	1.5 (+/-0.7) μM
Nephrotoxicity					
	Normal Range	Control	RDM-B	CDDP	CDDP+RDM-B
Urea	2.86-11.78 mM	6.4 (+/-1) mM	7.2 (+/-0.5) mM	5.55(+/-0.55) mM	10.66 (+/-3) mM
Creatinine	3.42-15.39 μM	9.5 (+/-5.5) μM	7.5 (+/-0.5) μM	13 (+/-1) μM	15 (+/-3) μM
Electrolytes					
	Normal Range	Control	RDM-B	CDDP	CDDP+RDM-B
Chloride	88-110 mM	115 (+/-2) mM	115.5 (+/-0.5) mM	115 (+/-1) mM	109.5 (+/-4.5) mM
Patassium	3.4-5.5 mM	4.65 (+/-0.35) mM	4.25 (+/-0.35) mM	4.3 (+/-0) mM	4.15 (+/-0.15) mM
Sodium	140-160 mM	151.5 (+/-0.5) mM	153.5 (+/-15) mM	150.5 (+/-0.5) mM	150 (+/-1) mM

Figure S1. A: Mouse body weight variations in the xenograft models of lung (A549) and ovarian (NIH:OVCAR-3) cancers for the control group and the groups treated by CDDP without and with the combination with RDM-B (BV10). It was observed that CDDP alone induced significant body weight decreases, whereas RDM-B (BV10) alone induced no body weight decreases and its combination with CDDP resulted in no additional body weight changes (P>0.05). B: Acute toxicity analysis in mice: the hepatotoxicity (ALT, ALP, TBIL), nephrotoxicity (blood urea, creatinine), and electrolytes (Na, K, Cl) were measured from the blood samples collected from the mice at 24 hours after the last treatment of the NIH:OVCAR-3 model, where ALP = lkaline phosphatase, ALT = alanine aminotransferase, and TBIL = total bilirubin.

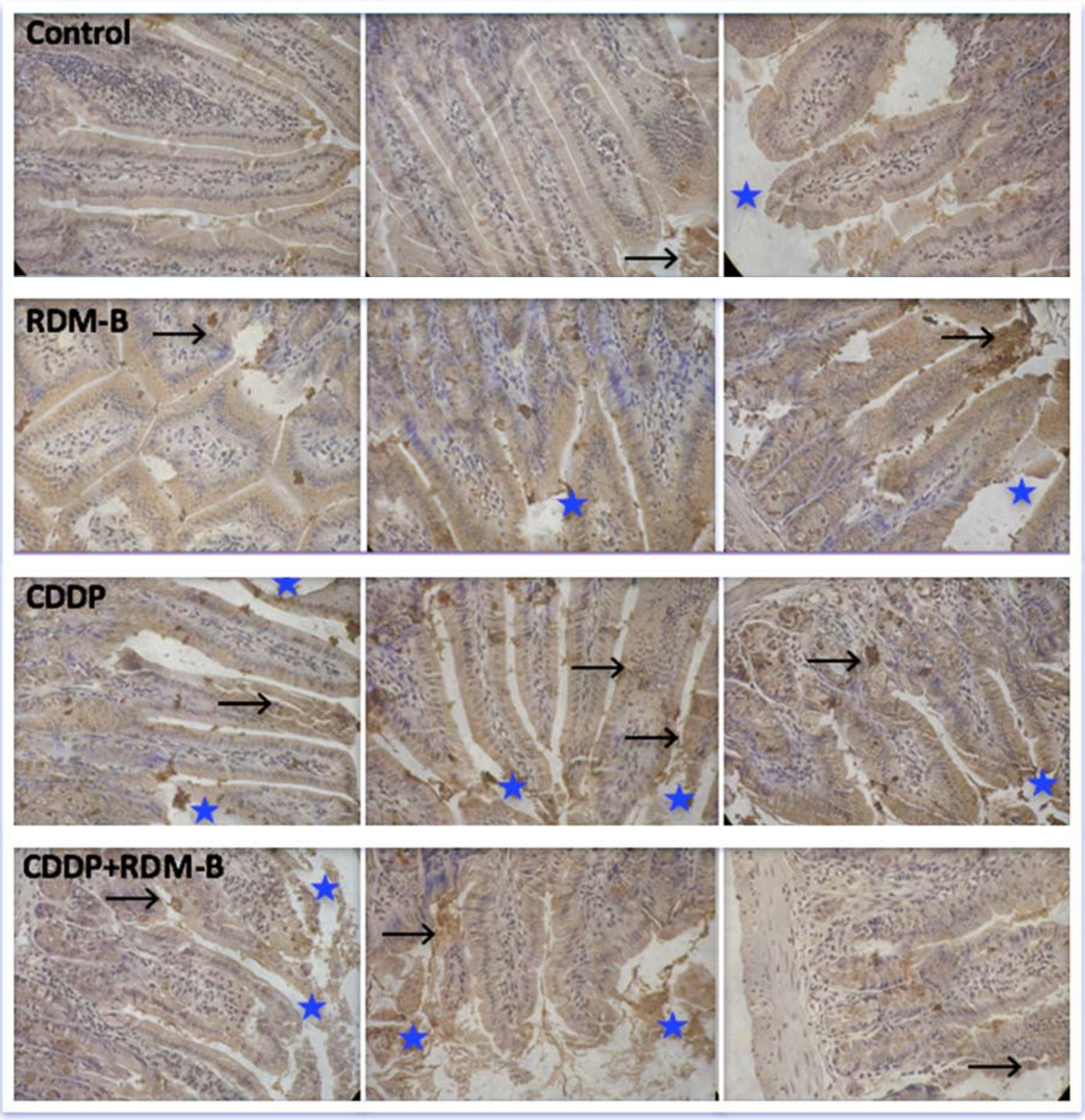

Figure S2. Representative TUNEL pictures of gut sections from NIH:OVCAR-3 tumor-bearing mice receiving different treatments. Black arrows and blue stars are used to indicate positively stained (apoptotic) cells and structure changes, respectively.
